# What Are the Best Materials to Enter into the Composition of Temporary Fillings to Be Retained for a Minimum of Three Years?

**Published:** 1893-06

**Authors:** L. Ashley Faught


### WHAT ARE THE BEST MATERIALS TO ENTER INTO THE COMPOSITION OF TEMPORARY FILLINGS TO BE RETAINED FOR A MINIMUM OF THREE YEARS?[1]

BY L. ASHLEY FAUGHT, D.D.S.

In the discussion of this question it is of first importance to understand alike and aright the meaning and use of the term "temporary." The word is defined by Webster as "lasting for a time only; existing or continuing for a limited time." In dentistry, its use as applied to filling-material is to express all that is opposed to the meaning of the term "permanent," and the testimony of practical experience is that quite frequently fillings, inserted with the idea of their permanency for a minimum of five years, often prove evanescent and fail of their purpose long before the expiration of that period. What is true of carefully-inserted work is found to be

---

[1] The remarks made at the February meeting of the Odontological Society of Pennsylvania by Dr. Faught were condensed in the regular report. At his request, and in justice to him, they are here presented in full.—Ed.

not less true of service rendered with the idea of even a minimum of three years. The speaker therefore conceives that it is impossible to speak of any filling as being either permanent or temporary, since varying conditions render it impossible to name with scientific accuracy the limited time of its continuance. It is time that the profession of dentistry ceased the use of the words temporary and permanent as related to filling-materials, except possibly as the word temporary is used, according to the definition of Dr. Ottolengui, to indicate a filling placed to cover dressings used in medication of the teeth, or inserted to tide over a few days until more convenient to fill, or to force the gums away from cavity borders, or for strictly temporary purposes. The treatment of dental caries by the insertion of filling-material is to restore lost tissue and to exclude deleterious influences which would irritate the pulp or contrive the destruction of the unnaturally-exposed soft tissue of the tooth. It is unscientific and absolutely impossible to prognosticate the duration of the efficacy of this service. Which of the many materials at our command should be used in any given case cannot be determined except by the operator after a careful consideration of the pathological conditions presenting and likely to surround the filling in its service. We may, as we have this evening, discuss with profit the chemical and mechanical qualities which any material has in itself to resist disintegration, but no statement can scientifically be made as to the length of time these qualities will remain inherent, when we add the element of their use under conditions of which it is impossible to know anything. These views are submitted not by way of cavil, or evasion, or criticism of the framing of the topic for discussion; but because, considering the fact that reports of these discussions are to become an epitome and consensus of the mind of the profession at the Columbian Dental Meeting, it is well not to give any foundation to the present too prevailing opinion of the public that fillings are warranted to last a specified time, as shoemakers guarantee a pair of shoes.

